# Integrating Generative AI Into Clinical Reasoning Education for Medical Students: Mixed Methods Study

**DOI:** 10.2196/94951

**Published:** 2026-08-13

**Authors:** Cheng-Heng Liu, Yu-Ting Chen, Chiun Hsu, Huey-Ling Chen, Chih-Wei Yang

**Affiliations:** 1 Department of Medical Education, National Taiwan University Hospital, No. 7, Zhongshan South Road, Zhongzheng District, Taipei City, 100, Taiwan, 886 223123456 ext 61426; 2 Department of Emergency Medicine, National Taiwan University Hospital, Taipei City, Taiwan; 3 Department of Living Sciences, National Open University, New Taipei City, Taiwan; 4 Graduate Institute of Medical Education and Bioethics, National Taiwan University College of Medicine, Taipei City, Taiwan; 5 Department of Medical Oncology, National Taiwan University Cancer Center, Taipei City, Taiwan; 6 Department of Pediatrics, National Taiwan University Hospital, Taipei City, Taiwan

**Keywords:** large language models, clinical reasoning, AI literacy, prompt engineering, medical education, generative AI, diagnostic decision-making

## Abstract

**Background:**

Generative AI (GenAI) is increasingly integrated into clinical learning and practice. However, medical students often lack the competencies required for safe and critical use, including prompt design, output verification, and recognition of limitations. Educational interventions that integrate GenAI with clinical reasoning frameworks remain limited.

**Objective:**

This study evaluated a structured, theory-informed workshop integrating GenAI, prompt engineering, and clinical reasoning education to enhance medical students’ self-perceived AI literacy and collaborative learning attitudes, and to assess whether patient-centered orientation changed following intensive AI exposure.

**Methods:**

We conducted a single-group, pre-post, explanatory sequential mixed methods study with fifth-year medical students enrolled at an academic medical center between April 2024 and May 2025. The 3-hour workshop comprised 6 modules integrating clinical reasoning, cognitive-bias awareness, verification-oriented GenAI use, and hands-on prompt engineering and centered on ChatGPT (OpenAI). Quantitative outcomes were self-reported measures from a 20-item self-report AI-literacy questionnaire adapted from the Meta AI Literacy Scale and were examined with exploratory and confirmatory factor analysis, the 6-item Patient-Practitioner Orientation Scale-Short, and a modified Collaborative Learning Attitude Scale (CLAS). Pre-post change was assessed using 2-tailed paired *t* tests with Benjamini-Hochberg correction and Cohen *d*; the Patient-Practitioner Orientation Scale-Short and CLAS were available for a subsample (n=46). Qualitative data from 6 interviews and 17 reflective narratives were analyzed using reflexive thematic analysis and integrated with the quantitative findings.

**Results:**

Among 150 eligible students, 139 (92.7%) completed paired AI-literacy assessments. Self-perceived AI literacy improved across all domains (Cohen *d*=0.69‐0.93, all *P*<.001; all remaining significant after false discovery rate correction), and collaborative learning attitudes increased substantially (*d*=0.94, *P*<.001). Patient-centered orientation showed no significant change (*d*=0.03); however, baseline scores were concentrated at the favorable end of the scale (a floor/restricted-range effect), and the subsample analysis was underpowered (minimum detectable *dz*=0.42), so this null result is inconclusive rather than evidence of unchanged orientation. Gains did not differ by sex or across the sequential cohorts. Reflexive thematic analysis (interviews: n=6; reflections: n=17) identified five themes and one emergent theme describing a shift toward verification-oriented GenAI use: (1) understanding GenAI capabilities and limitations, (2) prompt-engineering skill development, (3) calibrated trust through verification, (4) GenAI-supported communication and collaboration, and (5) ethical considerations, with emerging reconceptualization of professional identity.

**Conclusions:**

A brief, theory-informed educational intervention integrating GenAI with clinical reasoning was associated with medium-to-large improvements in self-perceived AI literacy and collaborative attitudes. No detectable change in patient-centered orientation was observed; however, this finding should be interpreted as inconclusive, given measurement and power constraints. Embedding verification practices within clinical reasoning frameworks may offer a scalable approach for preparing physicians for responsible human-AI collaboration. Future studies should incorporate comparative designs, performance-based assessments, and longitudinal follow-up.

## Introduction

Generative AI (GenAI), particularly large language models (LLMs), has rapidly transitioned from a speculative concept to a widely used clinical technology, with increasing relevance for medical education. Emerging evidence indicates that LLM-enabled systems may contribute to improvements in patient safety and quality of care, particularly in knowledge-intensive and documentation-heavy tasks [1]. Reviews of AI in health care have attributed this momentum to rapid advances in data availability and natural language processing, which have enabled increasingly sophisticated support for knowledge-intensive clinical tasks [2]. This rapid adoption has prompted growing interest in how future physicians should be prepared to engage with AI-enabled clinical environments, and recent frameworks have begun to define the digital and AI competencies expected of medical graduates [3].

Across health care settings, LLM-enabled systems are increasingly used to support clinical documentation and patient-facing communication, raising questions about how clinicians should incorporate AI-generated suggestions into reasoning and decision-making [1]. Experimental diagnostic vignettes further demonstrate their potential: in controlled comparisons, GenAI systems have matched or outperformed physicians on structured case assessments [4,5]. However, the evidence is mixed, and high-quality outputs do not necessarily reflect trustworthy reasoning processes. Systematic comparisons show variable performance across clinical decision-support tasks [6], and randomized trials indicate that simply providing clinicians with access to LLMs does not consistently improve diagnostic accuracy compared with conventional information resources [5]. Risks including hallucinations, embedded biases, and opaque reasoning may compromise patient safety if AI outputs are accepted uncritically [1,7,8]. Together, these findings underscore the importance of deliberate human-AI collaboration strategies rather than passive tool adoption.

Within medical education, clinical reasoning is conceptualized as a dynamic interplay between rapid pattern-recognition processes (System 1) and slower, analytical verification processes (System 2) [9]. A substantial body of research on cognitive bias describes how anchoring, premature closure, and related heuristics can derail both modes of reasoning [10]. Introducing GenAI into clinical learning environments without explicit instructional scaffolding may exacerbate these vulnerabilities by encouraging premature acceptance of AI-generated suggestions, thereby weakening reflective reasoning rather than strengthening it.

Despite growing enthusiasm for AI among medical trainees, surveys and needs assessments consistently indicate substantial gaps in preparedness for responsible and effective use. Learners report limited competence in prompt design, bias appraisal, and ethical boundary setting when interacting with GenAI systems [11,12], and a recent multi-institutional study confirms that positive student attitudes coexist with persistent concerns and unmet training needs [13]. Scholars have cautioned that uncritical reliance on AI tools may lead to a “lost cognitive perspective,” in which clinicians gradually offload essential reasoning processes to algorithms [8]. Together, these concerns highlight the need for medical curricula to move beyond passive exposure to AI technologies and toward structured, theory-informed educational interventions that integrate GenAI within established clinical reasoning frameworks.

To address this educational gap, we designed a 3-hour, 6-module workshop for fifth-year medical students that integrates hands-on GenAI prompt-engineering exercises with dual-process clinical reasoning, cognitive bias awareness, and explicit verification strategies. Rather than positioning AI as a shortcut to answers, the workshop frames GenAI as a powerful but fallible tool requiring contextual judgment and reflective oversight. The intervention was embedded within an existing clinical rotation to maximize ecological validity and align AI use with authentic case-based learning. A defining feature of this approach is its explicit intent to cultivate AI competencies alongside, rather than at the expense of, humanistic clinical values, treating patient-centered orientation not as a target for improvement, but as a benchmark for educational safety.

The primary objective of this study was to evaluate whether participation in this workshop was associated with improvements in medical students’ self-perceived AI literacy across knowledge, application, evaluation, and ethics domains. Secondary objectives were to examine changes in collaborative learning attitudes, conceptualized as openness to human-AI collaboration, and to assess whether patient-centered orientation changed following intensive exposure to AI tools. Using a mixed methods design, we sought not only to quantify changes in self-reported competencies but also to explore learners’ experiences and perceptions in order to generate meta-inferences about mechanisms of learning and professional adaptation in AI-augmented clinical reasoning education.

## Methods

### Study Design

We conducted a mixed methods educational evaluation using an explanatory sequential design (also termed sequential explanatory), in which a quantitative pre-post outcome phase was followed by a qualitative phase intended to help explain and contextualize the quantitative findings. The quantitative outcome data were collected immediately before and after the workshop, assessing changes in self-perceived AI literacy, collaborative learning attitudes, and patient-centered orientation; the qualitative interviews and reflective narratives were then collected in a subsequent phase to examine how students described their reasoning processes, trust calibration, and professional adaptation when using GenAI. The 2 strands were integrated at the interpretation stage.

Given the curricular constraints of a required clinical rotation, a single-group pre-post design was adopted to evaluate immediate educational impact. This design does not include a comparator group and therefore does not permit causal inference; findings are interpreted as changes associated with participation rather than as effects attributable solely to the intervention.

The study adhered to the GRAMMS (Good Reporting of a Mixed Methods Study) guidelines [14] (Checklist 1) to ensure transparency and rigor in the integration of qualitative and quantitative data.

### Setting and Participants

The study was conducted during the 2024 to 2025 academic year at the National Taiwan University College of Medicine. All 150 fifth-year undergraduate medical students enrolled in a required clinical rotation were invited to participate. The educational intervention was embedded within the existing curricular schedule, and participation in the research component was voluntary. Between April 2024 and May 2025, students participated in sequential cohorts of 11 to 13 students per session (13 sessions in total). Students were informed that opting out of research would not affect academic standing. To reduce cross-cohort contamination, students were reminded not to share detailed workshop materials with peers who had not yet attended. Taiwan’s medical education follows a 6-year undergraduate model; fifth-year students are therefore in their first year of formal clinical training. No structured AI-related curriculum had been offered during their preclinical years (years 1‐4), and the 2024 to 2025 academic year coincided with a period of rapid mainstream adoption of GenAI tools, making this workshop a timely and contextually relevant intervention for most participants. Participant demographic characteristics, including sex distribution and prior GenAI experience, are summarized in Table 1.

**Table 1.** Participant characteristics.

Variable and category	Participants, n (%)
Sex^a^	
Male	106 (76.3)
Female	33 (23.7)
Prior GenAI^b^ use	
Yes	138 (99.3)
No	1 (0.7)
GenAI use frequency^c^	
<1 day/week	17 (12.3)
About 1 day/week	21 (15.2)
2‐4 days/week	59 (42.8)
Almost daily	41 (29.7)

a One participant who selected “Other” for sex was included in the female category for reporting purposes.

b GenAI: generative AI.

c One participant who selected “Other” for frequency was excluded (n=138).

### Educational Intervention

The intervention consisted of a 3-hour, small-group workshop organized into 6 sequential instructional modules (approximately 25 min each) and delivered in an interactive format. The 6 modules accounted for 150 minutes of instructional time. An additional 20 minutes were allocated for preworkshop and postworkshop questionnaire completion, with the remaining 10 minutes used for introductions, transitions between modules, and a concluding wrap-up discussion. The 6 modules addressed the following topics: (1) Technology-Assisted Clinical Decision-Making; (2) Clinical Reasoning and Cognitive Biases; (3) GenAI and Prompt Engineering; (4) Critical Appraisal and Evidence Verification; (5) Patient-Centered Decision-Making with AI; and (6) Synthesis and Action Planning. Instructional strategies included brief didactic segments, clinical vignette analysis, structured prompt-engineering exercises, role-play exercises emphasizing patient-centered communication, and guided verification steps designed to support reflective human-AI collaboration.

The workshop centered on ChatGPT (OpenAI) as the primary GenAI platform; over the enrollment period (April 2024 to May 2025) this spanned the GPT-4 and GPT-4o model generations, and students were free to use other publicly available GenAI tools according to task demands. In module 4, students compared outputs from ChatGPT with those from domain-specific, evidence-based clinical reference tools (UpToDate and DynaMed) to reinforce the need for verification and contextual judgment. All activities used fictionalized clinical vignettes; students were instructed not to input identifiable patient information into GenAI tools.

An overview of module learning objectives and key activities is provided in Multimedia Appendix 1.

### Data Collection

To evaluate the effectiveness of the workshop, we administered 3 established instruments using a pre-post design to assess participants’ self-perceived AI literacy, patient-centered orientation, and collaborative learning attitudes.

AI literacy was assessed as self-perceived competence, using a 20-item self-report questionnaire adapted from the Meta AI Literacy Scale [15] and informed by an established AI literacy framework [16]; hereafter, this outcome is referred to as self-perceived AI literacy. Items were selected for contextual relevance to clinical reasoning, with minimal wording modification for medical education applicability. The questionnaire was translated into Mandarin Chinese and reviewed by bilingual medical education faculty for semantic equivalence. The final instrument assessed 4 domains, rated on a 5-point Likert scale (1=“strongly disagree” to 5=“strongly agree”): (1) Knowing and Understanding AI, (2) Applying AI, (3) Evaluating AI Applications, and (4) AI Ethics.

To examine the structure of the adapted instrument, exploratory factor analysis was conducted on pretest data (n=139) using principal axis factoring with Promax rotation. The Kaiser-Meyer-Olkin measure of sampling adequacy was 0.904 (excellent), and Bartlett test of sphericity was significant (*χ*²_190_=2504.2, *P*<.001). Parallel analysis suggested a 2-factor solution (a general competence factor and an ethics-awareness factor), whereas the theory-guided 4-factor solution explained 71.2% of total variance. The complete factor-loading matrices for both the 2-factor and 4-factor solutions are provided in Multimedia Appendix 2. To further examine the retained structure, a confirmatory factor analysis (CFA) of the 4-factor model was conducted on posttest data using maximum-likelihood estimation. Because this CFA drew on the same participants as the exploratory factor analysis, it does not constitute an independent validation of the factor structure. The 4 conceptual domains were highly intercorrelated (latent correlations up to *r*=0.93), and the model showed only modest fit (comparative fit index=0.90, Tucker-Lewis index=0.89, root-mean-square error of approximation=0.10, standardized root-mean-square residual=0.07). We therefore retain the 4-domain organization on theoretical and content-validity grounds rather than as a strongly discriminated empirical model; given the sample size, the high interfactor correlations, and the nonindependence of the exploratory and confirmatory analyses, these structural analyses are interpreted descriptively (see *Limitations* section). Internal consistency was acceptable to excellent (Cronbach α=0.77‐0.96 across domains; total α=0.93). Given the adaptation and translation, findings are interpreted as context-specific rather than as formal cross-cultural validation.

Patient-centered attitudes were assessed using the 6-item Patient-Practitioner Orientation Scale-Short (PPOS-D6) [17,18]. Items are rated on a 5-point Likert scale (1=strongly disagree to 5=strongly agree), with lower scores indicating greater patient-centered orientation. The PPOS-D6 has demonstrated acceptable psychometric properties in prior validation studies. Owing to a logistical constraint, this measure was administered only in the final 5 cohorts (January-May 2025), yielding complete pre-post data for 46 participants; analyses for this outcome are therefore interpreted cautiously, given the reduced and potentially nonrepresentative sample (see *Results* and *Limitations* sections). Collaborative learning attitudes were measured using 6 items adapted from the Collaborative Learning Attitude Scale (CLAS) [19,20], selected a priori based on item relevance; as with PPOS-D6, complete pre-post data were available for the same subset of participants (n=46). All questionnaires were translated into Mandarin Chinese and proofread by the author team prior to administration.

In this subsequent qualitative phase, data were obtained through semistructured interviews (n=6) and written reflective narratives (n=17) following the workshop. Participants were purposively sampled for interviews to reflect variation in baseline AI familiarity. The adequacy of the qualitative dataset was appraised using the concept of information power across the 5 dimensions described by Malterud et al [21]. The study aim was narrow, focusing on students’ experiences of one structured workshop. Sample specificity was high: all participants were fifth-year students who had completed the workshop. The study applied established theory, namely dual-process theory and reflexive thematic analysis. Dialogue quality was supported by the instructor-researchers’ familiarity with the workshop context, with the attendant positionality addressed under *Data Analysis*. Finally, the cross-case analysis drew on 2 complementary data types, with 17 written reflective narratives broadening the corpus beyond the six interviews. With reference to pragmatic data-adequacy benchmarks for interview studies [22], we judged this corpus sufficient for the descriptive, workshop-specific aims of this phase, while acknowledging the modest number of interviews (see *Limitations* section). The interview guide is available from the corresponding author upon reasonable request.

### Data Analysis

Quantitative data were analyzed using IBM SPSS Statistics for Windows (version 26.0; IBM Corp) and Python (SciPy, statsmodels, factor_analyzer, and semopy). Two-tailed paired-sample *t* tests were conducted to examine pre-post differences in outcome measures, and effect sizes were calculated using Cohen *d*. To account for multiple comparisons across the subscale pre-post tests, *P* values were adjusted using the Benjamini-Hochberg false discovery rate procedure [23], with Bonferroni correction reported for comparison; statistical significance was defined as *P*<.05. To assess the representativeness of the PPOS-D6/CLAS subsample, baseline characteristics (sex, prior GenAI use, baseline self-perceived AI literacy, and GenAI use frequency) were compared between the subsample and the remaining participants using the Welch *t* test, the Mann-Whitney *U* test, and the Fisher exact test as appropriate. A sensitivity power analysis was conducted for the PPOS-D6 total score. As an exploratory analysis, we examined whether gains in self-perceived AI literacy were moderated by sex and whether baseline scores or pre-post gains varied across the sequential cohorts (Spearman rank correlation with chronological cohort order). Given the single-group design, all analyses are interpreted as associations with participation rather than causal effects.

Qualitative data from interviews and reflective narratives were analyzed using reflexive thematic analysis, following Braun and Clarke [24-26]. An inductive coding approach was adopted. Two researchers independently familiarized themselves with the data, generated initial codes, and developed candidate themes, which were refined through iterative reflexive discussion; coding disagreements were resolved through this consensus process rather than by interrater reliability statistics, consistent with a reflexive approach. To address trustworthiness, we supported credibility through triangulation across interviews and reflective narratives; dependability through a consistently applied coding procedure with consensus resolution of discrepancies; confirmability through independent dual coding and explicit reflexivity; and transferability through thick contextual description of the setting and participants. As a positionality statement, the authors reflexively acknowledge their dual roles as both workshop instructors and researchers and remain attentive to how this positionality could shape data generation and interpretation; we sought to mitigate it through independent coding and reflexive discussion. Representative quotations were translated from Mandarin Chinese and verified by bilingual authors.

### Mixed Methods Integration

Integration occurred at the interpretation and meta-inference level. Quantitative changes in self-perceived AI literacy and collaborative attitudes were examined alongside qualitative themes describing development of prompt-engineering competence, verification-oriented reasoning, calibrated trust, and professional identity adaptation. Joint interpretation enabled identification of 3 integrated learning dimensions: (1) technical AI literacy acquisition, (2) verification-oriented reasoning development, and (3) professional and ethical adaptation. This integrative approach allowed the qualitative findings to explain and elaborate the self-reported quantitative improvements. All authors participated in the integration process.

### Ethical Considerations

This educational study was approved by the institutional review board of the National Taiwan University Hospital (institutional review board number 202403120RINA). All participants provided informed consent electronically prior to study participation. Participation in surveys and qualitative feedback activities was voluntary. Although the workshop was a required curricular activity, students who opted out of the research component received identical educational content. To protect privacy and confidentiality, survey responses were collected without personal identifiers, and baseline characteristics (eg, sex and GenAI use) were collected. Qualitative data were deidentified prior to analysis. Participants did not receive financial compensation for their involvement in the study.

## Results

### Participant Flow and Baseline Characteristics

Among 150 eligible fifth-year medical students, 139 (92.7%) completed both preintervention and postintervention surveys for AI literacy and were included in the primary quantitative analysis. The sample comprised 106 (76.3%) male students and 33 (23.7%) female students, a distribution consistent with the sex composition of the medical class at our institution. Nearly all respondents (138/139, 99.3%) reported prior experience with GenAI, with the most common usage frequency being 2 to 4 days per week (59/138, 42.8%). Owing to a logistical limitation, the PPOS-D6 and the CLAS were administered only to 46 participants from the final 5 cohorts (January-May 2025); these analyses are therefore reported separately, and the representativeness of this subsample is examined below. Qualitative data were obtained from 6 semistructured interviews and 17 written reflections.

### Self-Perceived AI Literacy

Significant improvements were observed across all self-perceived AI literacy domains and in the total score (Table 2). The mean total score increased from 3.57 (SD 0.64) preintervention to 4.23 (SD 0.54) postintervention (*t*_138_=11.55, *P*<.001), corresponding to a large effect size (Cohen *d*=0.98, 95% CI 0.77‐1.19). Effect sizes of domain-level changes ranged from 0.69 to 0.93 (Knowledge: *d*=0.90, 95% CI 0.70‐1.10; Application: *d*=0.69, 95% CI 0.49‐0.88; Evaluation: *d*=0.93, 95% CI 0.73‐1.13; Ethics: *d*=0.79, 95% CI 0.59‐0.99), with the largest changes observed in the Knowledge and Evaluation domains. All domain improvements remained significant after Benjamini-Hochberg correction for multiple comparisons (all adjusted *P*<.001; the same conclusion held under the more conservative Bonferroni correction).

**Table 2.** Self-perceived AI literacy pre-post comparison (n=139).

Domain	Pretest, mean (SD)	Posttest, mean (SD)	*t* (*df*=138)	*P* value	Cohen *d* (95% CI)
Knowledge	3.72 (0.65)	4.28 (0.53)	10.63	<.001	0.90 (0.70‐1.10)
Application	3.62 (0.81)	4.19 (0.62)	8.09	<.001	0.69 (0.49‐0.88)
Evaluation	3.45 (0.82)	4.23 (0.61)	10.91	<.001	0.93 (0.73‐1.13)
Ethics	3.48 (1.00)	4.24 (0.79)	9.27	<.001	0.79 (0.59‐0.99)
Total	3.57 (0.64)	4.23 (0.54)	11.55	<.001	0.98 (0.77‐1.19)

Internal consistency for the self-perceived AI literacy domains in this sample was acceptable to excellent, with Cronbach α values ranging from 0.77 to 0.96.

In an exploratory analysis, gains in self-perceived AI literacy did not differ by sex: total gains were near-identical for male and female students (+0.65 vs +0.64; Welch *t*=0.14, *P*=.89, *d*=0.02; 106 male vs 32 female, with the single participant who selected “other” excluded from this analysis), and no domain showed a significant difference (all *P*≥.74), and baseline scores did not differ by sex (*P*=.48). Across the 13 sequential cohorts (n=138; one response with an unparseable timestamp was excluded), pre-post gains were unrelated to cohort order (Spearman ρ=–0.07, *P*=.39), and baseline self-perceived AI literacy was not significantly associated with cohort order (Spearman ρ=0.17, *P*=.05); cohort- and semester-level descriptive statistics are provided in Multimedia Appendix 2.

### Patient-Practitioner Orientation

Using the 6-item PPOS-D6, no significant pre-post differences were observed for the Sharing (*d*=–0.09, 95% CI –0.39 to 0.21), Caring (*d*=0.17, 95% CI –0.13 to 0.47), or Total (*d*=0.03, 95% CI –0.27 to 0.33) scores (Table 3). This null result should be interpreted in light of 2 constraints. First, the subsample (the final 5 cohorts) was not fully representative of the whole cohort: although comparable in sex distribution (Fisher exact *P*=.09) and prior GenAI use (*P*>.99), it had higher baseline self-perceived AI literacy (3.74 vs 3.48; Welch *t*=2.59, *P*=.01, *d*=0.42) and more frequent GenAI use (Mann-Whitney *P*<.001; both differences remained significant after Benjamini-Hochberg correction), consistent with its being composed of later cohorts. Second, because PPOS-D6 items are scored such that lower values indicate greater patient-centered orientation, the low baseline scores reflect an already strongly patient-centered orientation at entry: baseline total scores clustered at the favorable (low) end of the scale (mean 1.84, SD 0.50, maximum 2.83), no participant scored above the scale midpoint, and responses occupied only about 46% of the available range (a floor/restricted-range effect; Multimedia Appendix 2). A sensitivity power analysis indicated that, with 46 participants, the study could detect only medium-or-larger within-person change (minimum detectable *dz*=0.42 at 80% power; observed *dz*=0.03, corresponding to achieved power of approximately 5%). The nonsignificant PPOS-D6 result is therefore inconclusive on both representativeness and measurement grounds rather than evidence that orientation was unchanged.

**Table 3.** Patient-practitioner orientation pre-post comparison (n=46).

Dimension	Pre, mean (SD)	Post, mean (SD)	*t* (*df*=45)	*P* value	Cohen *d* (95% CI)
Sharing	1.94 (0.62)	1.88 (0.92)	–0.62	.54	–0.09 (–0.39 to 0.21)
Caring	1.75 (0.59)	1.84 (0.80)	1.12	.27	0.17 (–0.13 to 0.47)
Total	1.84 (0.50)	1.86 (0.78)	0.18	.86	0.03 (–0.27 to 0.33)

### Collaborative Learning Attitude

Scores on the modified CLAS increased significantly after the workshop (*t*_45_=6.39, *P*<.001). Mean CLAS total scores increased from 3.74 (SD 0.44) to 4.22 (SD 0.64), with a large effect size (*d*=0.94, 95% CI 0.61‐1.27); this improvement remained significant after Benjamini-Hochberg correction. As this outcome was assessed in the same subsample of 46 participants, the same representativeness caveat applies.

### Qualitative Findings

Thematic analysis of semistructured interviews (n=6) and written reflective narratives (n=17) identified 5 major themes and 1 emergent theme from reflections. Table 4 summarizes themes and representative quotes. Additional quotes, subthemes, and the 4-stage learning trajectory are provided in Multimedia Appendix 1. Themes encompassed the following:

1. Understanding GenAI Capabilities and Limitations: Students described GenAI as useful for information synthesis and differential diagnosis expansion, while recognizing its limitations in contextual judgment and localization. Several participants emphasized the probabilistic nature of outputs and the necessity of contextual interpretation.
2. Development of Prompt-Engineering Skills: Participants reported progression from generic queries to structured, context-rich prompts. They described learning to specify task framing, request justification, and iteratively refine prompts. Prompt engineering was increasingly conceptualized as a clinical skill analogous to structured history-taking.
3. Calibrated Trust Through Verification Practices: Students emphasized verification as a mandatory step. They reported cross-checking outputs against textbooks, guidelines, or supervisory input. Rather than treating AI as authoritative, learners described adopting a “calibrated trust” stance, adjusting reliance based on task complexity and stakes.
4. GenAI-Supported Communication and Collaboration: Participants viewed GenAI as helpful for translating medical terminology into lay language and generating alternative perspectives during group discussion. However, they consistently positioned empathy and relational care as distinctly human responsibilities.
5. Ethical and Educational Considerations: Students raised concerns about privacy, accountability, bias, and overreliance. Some expressed uncertainty regarding institutional guidance for AI use.

With regard to the emergent theme—Professional Identity Reconceptualization—reflective writings suggested evolving perceptions of the physician’s role in AI-augmented practice. Some students described transitioning from sole knowledge holders to “AI-augmented decision makers,” while others expressed ambivalence regarding dependency on technology.

**Table 4.** Summary of qualitative themes and representative quotes.

Theme	Key finding	Representative quote^a^
Understanding GenAI^b^ capabilities and limitations	GenAI was perceived as useful for knowledge integration but limited by context and localization.	“AI only sees the keywords we input; it cannot fully comprehend the complete situation.” (P-E)
Development of prompt-engineering skills	Students progressed from generic prompts to structured, context-rich prompting and task-based tool selection.	“You need to write better instructions, specifically telling it which references to use.” (P-D)
Calibrated trust through verification	Learners emphasized verification and deference to human clinical judgment when using GenAI outputs.	“The first step is always to verify or read through myself.” (P-A)
GenAI-supported communication and collaboration	GenAI supported translation of information for patients and teamwork, while empathy remained a human responsibility.	“Generative AI has the unique ability to quickly translate medical information into lay language.” (P-D)
Ethical and educational considerations	Students raised concerns about privacy, accountability, and overreliance on GenAI in learning.	“Because AI is so convenient, we might lose our ability to research original textbooks.” (P-E)
Emergent theme: professional identity reconceptualization	Reflections described shifts in perceived physician roles and future practice in AI-augmented care.	“We are standing at the starting point of this transformation.” (RH)

a Participant IDs beginning with “P” denote interview participants, and IDs beginning with “R” denote authors of written reflective narratives. All quotations were translated from Mandarin Chinese.

b GenAI: generative AI.

### Mixed Methods Integration

Integration of quantitative and qualitative findings revealed three interconnected dimensions of learning:

1. Technical Competence Development: Quantitative improvements in self-perceived AI literacy were reflected qualitatively in students’ descriptions of improved prompt design and critical appraisal skills.
2. Verification-Oriented Reasoning: Gains in the Evaluation and Ethics domains aligned with qualitative accounts of calibrated trust and structured verification practices.
3. Professional Adaptation in Human-AI Contexts: Increased collaborative learning attitudes corresponded with qualitative themes of human-AI teaming and evolving professional identity.

Collectively, these integrated findings suggest that the intervention was associated not only with increased self-perceived AI literacy but also with shifts in reasoning processes and conceptualizations of human-AI collaboration.

## Discussion

### Principal Results

To our knowledge, this study is among the first mixed methods investigations to examine how structured prompt engineering and AI literacy training can be integrated into undergraduate clinical reasoning education. The findings suggest that effective use of GenAI depends not only on access to advanced models but also on learners’ ability to critically direct, evaluate, and contextualize AI-generated outputs.

In this structured GenAI-integrated clinical reasoning workshop, participation was associated with medium-to-large improvements in self-perceived AI literacy across knowledge, application, evaluation, and ethics domains, alongside substantial gains in collaborative learning attitudes. Patient-centered orientation did not demonstrate measurable short-term change; as detailed below and in the *Limitations* section, this null result is inconclusive, given the restricted baseline range and limited statistical power rather than firm evidence of stability. Qualitative findings complemented these results by revealing a developmental trajectory from passive tool use toward verification-oriented, context-aware engagement with GenAI systems, with students beginning to reconceptualize their professional roles within AI-augmented clinical environments.

Because all quantitative outcomes were self-reported, the observed improvements reflect self-perceived competence and confidence rather than demonstrated performance, and may be subject to response-shift bias, social desirability, and demand characteristics, particularly because the instructors also served as assessors. We therefore interpret the quantitative gains as changes in self-perceived AI literacy and recommend that future studies corroborate them with objective, behaviorally anchored measures, such as structured AI-use vignettes, AI-integrated objective structured clinical examination stations, and rubric-based assessment of prompt quality. With this caveat, the integration of quantitative and qualitative data indicates that self-perceived AI-literacy acquisition was accompanied by qualitatively described shifts in reasoning stance and professional orientation.

### Comparison With Prior Work

A central educational concern in AI integration is automation bias, the tendency to overrely on automated outputs without adequate scrutiny [27]. In this study, improvements in the Evaluation and Ethics domains were mirrored by qualitative descriptions of deliberate verification, with students cross-checking AI outputs against trusted sources and supervisory judgment. This pattern aligns with the concept of calibrated trust in human-AI systems, whereby appropriate reliance is dynamically adjusted to task context and risk [28]. Crucially, verification was treated not as an ancillary safeguard but as a structured component of reasoning instruction: by situating GenAI within dual-process theory, learners were encouraged to treat AI outputs as hypotheses for analytical evaluation (System 2) rather than as definitive answers. This framing may mitigate the “lost cognitive perspective” described in prior literature [8] and supports a pedagogical shift from “AI familiarity” toward verification-oriented AI literacy. Importantly, our findings suggest that AI literacy should not be equated with mere familiarity or frequency of AI use. Rather, meaningful AI literacy requires the ability to critically interrogate outputs, recognize uncertainty, identify hallucinations, and determine when AI recommendations should or should not influence clinical decisions. Consistent with calls for critical AI literacy in medical education [8], students increasingly recognized AI’s strengths in pattern recognition and retrieval alongside its limitations in contextual judgment, suggesting that explicit discussion of model capabilities and failure modes should be a core curricular element. From a cognitive load perspective, AI can reduce the extraneous load of information retrieval [29], but this convenience may also invite cognitive offloading; mandating verification positions AI as a scaffold for, rather than a substitute for, the development of clinical reasoning [30].

Learners also progressed from generic prompting to structured, context-rich queries, describing a 4-stage trajectory of passive reception, basic engagement, strategic application, and critical monitoring. This suggests that prompt engineering can be taught as a clinical skill analogous to structured history-taking, in which learners provide organized information, request justification, and iteratively refine queries; the observed Application-domain gain (*d*=0.69) is consistent with this interpretation, and hands-on practice anchored in authentic scenarios and scaffolded by instructor feedback appeared essential to its development.

The substantial increase in collaborative learning attitudes (*d*=0.94), together with qualitative accounts of AI as a collaborative partner, valued for translating technical information into lay language while empathy and relational continuity remained firmly human responsibilities, aligns with the centaur model of human-AI collaboration, in which combined human and machine intelligence outperforms either entity alone [31]. Reflective writings further suggested emerging professional identity adaptation [32], with students moving from sole knowledge holders toward “AI-augmented decision makers”; some voiced concern about overreliance (“we might lose our ability to research original textbooks”), while others described themselves as “standing at the starting point of transformation,” underscoring the need to integrate AI into professionalism and identity-formation curricula, not only technical training. In contrast, patient-centered orientation did not change detectably; given the baseline floor effect and limited statistical power, this is most consistent with, though it cannot confirm, the premise that AI literacy and patient-centeredness need not be antagonistic competencies when instruction explicitly integrates ethical reflection and communication practice [33]. Adequately powered longitudinal research with more sensitive measures is needed to determine whether GenAI–reasoning curricula influence or safeguard patient-centeredness over time.

### Educational Design Implications

Collectively, these findings suggest three interrelated dimensions for AI curriculum design (technical AI literacy, verification-oriented reasoning, and professional/sociotechnical adaptation) and four practical design principles for GenAI-integrated curricula: (1) embed AI activities within authentic clinical reasoning tasks to support skill transfer; (2) treat AI as powerful but fallible, with verification as a required step; (3) use small-group formats that mirror collaborative clinical environments; and (4) explicitly connect AI use to patient-centered communication and ethical responsibility. These principles align with broader calls for sociotechnical AI education and may support scalable curriculum development.

### Limitations

Several limitations warrant consideration. First, all quantitative outcomes were self-reported measures of perceived competence rather than objective performance, and may be affected by response-shift bias, social desirability, and demand characteristics, particularly because the instructors also served as assessors. Although the workshop focused on clinical reasoning, the study did not directly assess clinical reasoning performance. Therefore, it remains unclear whether improvements in self-perceived AI literacy translate into enhanced diagnostic reasoning or clinical decision-making. Future studies should incorporate objective, behaviorally anchored assessments, such as structured AI-use vignettes, AI-integrated objective structured clinical examination stations, script concordance tests, key-feature problems, or rubric-based evaluations of diagnostic reasoning and prompt quality. Second, the single-group pre-post design without a comparator group limits causal inference; observed changes are described as associated with participation rather than attributable solely to the intervention, and no comparator data were available for a sensitivity analysis. Third, the PPOS-D6 and CLAS analyses were limited to a subsample of 46 participants from the final 5 cohorts; this subsample had higher baseline self-perceived AI literacy and more frequent GenAI use than the remaining participants, limiting representativeness, and the study was powered to detect only medium-or-larger change in PPOS-D6 (minimum detectable *dz*=0.42), so the null PPOS-D6 result is inconclusive, particularly given a baseline floor/restricted-range effect reflecting an already strongly patient-centered orientation. In addition, the unbalanced sex distribution (106 male vs 32 female) limited the power of the sex-moderation analysis to detect small-to-moderate effects (minimum detectable *d*≈0.57), so the absence of sex differences should not be interpreted as evidence of equivalence. Fourth, although the 4-domain AI-literacy structure was retained on theoretical grounds, the domains were highly intercorrelated and a CFA showed only modest fit. Because both analyses relied on the same participants, the CFA cannot independently confirm the factor structure and may capitalize on sample-specific characteristics [34]. These structural analyses are therefore descriptive, and validation of the 4-domain structure in an independent sample is needed before it can be treated as confirmed. Fifth, data collection spanned 13 months during which public exposure to GenAI evolved and the underlying models advanced; although gains were unrelated to cohort timing, a nonsignificant change in baseline self-perceived AI literacy across cohorts, together with the possibility of informal cross-cohort sharing of materials, cannot be excluded. Finally, the study was conducted at a single academic medical center with high baseline AI exposure, and the qualitative corpus, while enriched by 17 reflective narratives, was based on six interviews; generalizability to other institutions or earlier-stage learners may therefore be limited.

### Conclusions

As GenAI tools continue to evolve and permeate clinical environments, medical education should move beyond passive exposure toward structured, theory-informed curricula that help future physicians harness AI’s potential responsibly. By interweaving technical skills with clinical reasoning and humanistic principles, medical education can prepare physicians who not only use AI but also guide its responsible implementation in patient care. This study indicates that a concise, theory-informed workshop was associated with improvements in self-perceived AI literacy and collaborative attitudes while explicitly emphasizing critical thinking and humanistic values. The remaining challenge lies in scaling such interventions while maintaining educational quality and addressing the ethical complexities associated with AI in medicine.

Future research should incorporate comparative designs, objective performance-based measures, and longitudinal follow-up to examine durability, behavioral transfer, and professional identity development in AI-augmented clinical environments, particularly as medicine moves toward more transparent and explainable AI systems [35].

## Supplementary material

10.2196/94951 Multimedia Appendix 1 Workshop module overview and extended qualitative findings.

10.2196/94951 Multimedia Appendix 2 Supplementary statistical tables.

10.2196/94951 Checklist 1 GRAMMS checklist.

## Abbreviations

CFA: confirmatory factor analysis
CLAS: Collaborative Learning Attitude Scale
GenAI: generative AI
GRAMMS: Good Reporting of a Mixed Methods Study
LLM: large language model
PPOS-D6: Patient-Practitioner Orientation Scale-Short
