# Supplementary material for: Integrating Generative AI Into Clinical Reasoning Education for Medical Students: Mixed Methods Study
Source: JMIR Med Educ. 2026 Aug 13;12:e94951. doi: 10.2196/94951 (PMC13472228; doi:10.2196/94951)
Supplement: Supplementary file 1 [file mededu-v12-e94951-s001.docx]

# **Workshop Module Overview and Extended Qualitative Findings**

## **Workshop Modules**

**Table A1. Overview of Workshop Modules, Learning Objectives, and Key Activities**

| Module | Focus and learning objectives | Key activities |
| --- | --- | --- |
| 1. Introduction to Technology-Assisted Clinical Decision-Making | Outline the historical evolution of decision-support tools; Compare rule-based systems versus LLMs; Identify opportunities and pitfalls of GenAI in acute care | Mini-lecture; real-world use-case polling; facilitated discussion |
| 2. Clinical Reasoning and Cognitive Biases | Review inductive/deductive pathways and dual-process theory; Recognize anchoring, premature closure, availability, and related biases; Map bias checkpoints into SOAP note workflow | Interactive concept-mapping; bias-spotting vignettes |
| 3. GenAI and Prompt Engineering | Explain LLM architecture, context windows, hallucination risks; Practice structured role-task-context prompting and iterative chaining | Hands-on vignette prompts in triads; live debrief |
| 4. Critical Appraisal and Evidence Verification | Apply PICO framing to validate AI-suggested diagnoses; Use checklists (accuracy, relevance, grade of evidence); Compare outputs from ChatGPT (OpenAI) versus domain-specific tools; Retrieve and appraise primary literature supporting AI output | Rapid evidence scavenger hunt; critical appraisal discussion; tool comparison exercise |
| 5. Patient-Centered Medical Decision-Making | Integrate AI suggestions with patient values/preferences; Practice shared decision-making role-plays using AI-generated handouts; Discuss trust calibration and explainability for lay audiences | Standardized-patient role-play; reflective peer feedback |
| 6. Summary of Core Concepts | Synthesize take-home messages; Draft personal action plans for responsible AI use; Identify next-step resources (eg, institutional policy, further MOOCs) | Fishbowl recap; exit ticket commitments |

## **Four-Stage Learning Trajectory in AI Interaction**

Across interviews and reflections, students described a four-stage trajectory in how they engaged with GenAI: (1) passive reception, (2) basic engagement, (3) strategic application, and (4) higher-order critical use. During passive reception, students reported limited understanding of AI capabilities and minimal prompting skills, often accepting vague outputs at face value. In basic engagement, students began refining prompts, questioning responses, and recognizing contextual limitations in clinical scenarios. The strategic application stage was characterized by tailoring prompts to vignette details, cross-checking AI-generated information against other resources, and integrating AI suggestions with their own clinical reasoning. Finally, a subset of students described higher-order critical use in which they actively monitored for hallucinations, weighed ethical and privacy implications, and reflected on their own dependence on AI tools.

## **Extended Representative Quotes by Theme**

**Table A2. Thematic Analysis of Medical Students’ Perceptions of GenAI in Clinical Reasoning**

| Theme | Sub-theme | Representative quotations |
| --- | --- | --- |
| Understanding GenAI characteristics | Knowledge integration | *“Beyond providing clinical information, I can inquire about basic medical sciences and pathophysiology, which would be difficult to obtain from a single textbook.”* (P-C) |
|  | Systematic reasoning | *“It can produce systematic and comprehensive differential diagnoses… listing all possibilities, even those with very low probability.”* (P-A) |
|  | Contextual limitations | *“AI only sees the keywords we input; it cannot fully comprehend the complete situation.”* (P-E) |
|  | Geographic specificity | *“GPT’s information comes from various regions and may not be specific to certain localities… some diseases are region-specific.”* (P-E) |
| Development of prompt-engineering skills | Initial experience | *“We weren’t familiar with prompting initially, so we simply asked it to act as a professional doctor and provide differential diagnoses based on given conditions.”* (P-B) |
|  | Strategic development | *“You need to write better instructions, specifically telling it which references to use.”* (P-D) |
|  | Structured input | *“It requires careful listing of patient events before providing them to GPT.”* (P-F) |
| Calibrated trust through verification | Deference to human judgment | *“I would consider the clinical instructor’s judgment more accurate because… they have a deeper understanding of Taiwanese patients or NTU Hospital patients.”* (P-F) |
|  | Systematic verification | *“The first step is always to verify or read through myself, ensuring the answer doesn’t contradict my understanding.”* (P-A) |
|  | Critiquing AI flaws | *“I still cannot fully connect textual fluency with accuracy.”* (P-C) |
| GenAI’s role in communication and collaboration | Information translation | *“Generative AI has the unique ability to quickly translate medical information into lay language… helping bridge professional communication gaps.”* (P-D) |
|  | Supportive tool | *“For me, it’s just an auxiliary tool. The final presentation and reporting of data still requires my own processing.”* (P-A) |
|  | Human-centered approach | *“It’s really like an assistant… I know it might fabricate things, so I always verify.”* (P-D) |
| Ethical and educational considerations | Legal concerns | *“If AI makes a diagnostic error… it’s unclear whether the legal responsibility falls on the hospital, the vendor, or the physician.”* (P-F) |
|  | Dependency risk | *“Because AI is so convenient, we might lose our ability to research original textbooks.”* (P-E) |
|  | Knowledge foundation | *“We should first establish our own knowledge structure… AI should only assist with unclear areas.”* (P-B) |

*Note: Participant IDs (P-A to P-F) refer to different participants. All quotations were translated from Mandarin Chinese.*

## **Emergent Theme: Professional Identity Reconceptualization**

Reflective writings highlighted students’ evolving views of professional identity in the AI era:

- *“I originally thought that medicine, as a profession requiring human interaction, would have relatively less contact with artificial intelligence.”* (RJ)
- *“If medical students can master this technology, they will be able to more efficiently diagnose diseases, write research, and even lead medical innovation in the future.”* (RH)
- *“This is a journey of medical and technological fusion, and we are standing at the starting point of this transformation.”* (RH)
- *“Perhaps soon, patients won’t come to consultations with Google information, but with ChatGPT’s treatment recommendations.”* (RE)

*Note: Reflection IDs (RJ, RH, RE) refer to different participants. All quotations were translated from Mandarin Chinese.*
