# Supplementary material for: Integrating Generative AI Into Clinical Reasoning Education for Medical Students: Mixed Methods Study
Source: JMIR Med Educ. 2026 Aug 13;12:e94951. doi: 10.2196/94951 (PMC13472228; doi:10.2196/94951)
Supplement: Supplementary file 2 [file mededu-v12-e94951-s002.docx]

## Supplementary statistical tables

This appendix provides the complete factor-loading matrices (two- and four-factor exploratory factor analysis), confirmatory factor analysis fit indices, cohort- and semester-level trends in self-perceived AI literacy, and the PPOS-D6 score distribution.

## Table S1. EFA two-factor solution, pre-test (n=139)

Principal axis factoring with Promax rotation. KMO = 0.904; Bartlett χ²(190) = 2504.2, P<.001. Variance explained: F1 41.3%, F2 25.1% (cumulative 66.4%). Bold = |loading| ≥ 0.30.

| Item | Theory domain | F1 (Competence) | F2 (Ethics) |
| --- | --- | --- | --- |
| 1 | Knowledge | **0.37** | **0.37** |
| 2 | Knowledge | **0.84** | −0.10 |
| 3 | Knowledge | **0.38** | **0.41** |
| 4 | Knowledge | **0.72** | −0.19 |
| 5 | Knowledge | **0.61** | −0.06 |
| 6 | Application | **0.92** | −0.15 |
| 7 | Application | **0.78** | 0.00 |
| 8 | Application | **0.89** | −0.13 |
| 9 | Application | **0.90** | −0.16 |
| 10 | Application | **0.90** | −0.09 |
| 11 | Evaluation | **0.72** | 0.04 |
| 12 | Evaluation | **0.65** | 0.23 |
| 13 | Evaluation | **0.69** | 0.25 |
| 14 | Evaluation | **0.76** | 0.17 |
| 15 | Evaluation | **0.68** | 0.12 |
| 16 | Ethics | −0.15 | **0.94** |
| 17 | Ethics | −0.20 | **0.98** |
| 18 | Ethics | −0.05 | **0.95** |
| 19 | Ethics | −0.04 | **0.92** |
| 20 | Ethics | −0.06 | **0.92** |

## Table S2. EFA four-factor solution, pre-test (n=139)

Principal axis factoring with Promax rotation. Variance explained: F1 24.8%, F2 20.9%, F3 9.2%, F4 16.3% (cumulative 71.2%). Bold = |loading| ≥ 0.30.

| Item | Theory domain | F1 | F2 | F3 | F4 |
| --- | --- | --- | --- | --- | --- |
| 1 | Knowledge | −0.01 | 0.04 | **0.89** | 0.09 |
| 2 | Knowledge | **0.30** | 0.01 | −0.04 | **0.62** |
| 3 | Knowledge | 0.06 | 0.09 | **0.83** | 0.07 |
| 4 | Knowledge | −0.13 | −0.10 | 0.22 | **0.80** |
| 5 | Knowledge | **−0.30** | 0.14 | 0.05 | **0.95** |
| 6 | Application | **0.40** | −0.06 | −0.02 | **0.59** |
| 7 | Application | **0.46** | 0.06 | −0.05 | **0.42** |
| 8 | Application | **0.33** | 0.02 | −0.11 | **0.68** |
| 9 | Application | **0.63** | −0.16 | 0.01 | **0.32** |
| 10 | Application | **0.64** | −0.01 | −0.16 | **0.40** |
| 11 | Evaluation | **0.58** | −0.12 | **0.32** | 0.08 |
| 12 | Evaluation | **0.81** | 0.00 | 0.29 | −0.19 |
| 13 | Evaluation | **0.85** | 0.06 | 0.18 | −0.14 |
| 14 | Evaluation | **0.97** | 0.06 | −0.02 | −0.11 |
| 15 | Evaluation | **0.88** | 0.12 | −0.22 | −0.02 |
| 16 | Ethics | −0.03 | **0.89** | 0.06 | 0.01 |
| 17 | Ethics | −0.06 | **0.96** | −0.02 | 0.02 |
| 18 | Ethics | 0.11 | **0.89** | 0.03 | −0.02 |
| 19 | Ethics | 0.08 | **0.89** | −0.01 | 0.03 |
| 20 | Ethics | 0.03 | **0.88** | 0.05 | 0.04 |

Note. The four conceptual domains are highly intercorrelated; Knowledge and Application items distribute across F1/F4, while Ethics (F2) and an Evaluation-led factor (F1) are the most clearly separated dimensions. This pattern is consistent with the parallel-analysis result favoring two factors and with the modest CFA fit (Table S3).

## Table S3. CFA fit indices — theory-based four-factor model, post-test (n=139)

Maximum-likelihood estimation; four correlated latent factors (Knowledge, Application, Evaluation, Ethics).

| Index | Value | Conventional threshold | Interpretation |
| --- | --- | --- | --- |
| χ²(164) | 393.3 (P<.001) | — | Significant (expected at this model size) |
| CFI | 0.903 | ≥.95 good; ≥.90 acceptable | Marginal |
| TLI | 0.887 | ≥.95 good; ≥.90 acceptable | Below .90 |
| RMSEA | 0.101 | ≤.06 good; ≤.08 acceptable | Exceeds .08 |
| SRMR | 0.070 | ≤.08 | Acceptable |
| GFI / AGFI / NFI | 0.846 / 0.821 / 0.846 | ≥.90 | Below threshold |

Note. Fit was modest, and latent inter-factor correlations were high (up to r=0.93). The four-domain structure is retained on theoretical and content-validity grounds; structural results are reported descriptively given the sample size (≈3:1 subjects-to-parameters) and the non-independence of the exploratory (pre-test) and confirmatory (post-test) data, which were provided by the same participants; the CFA therefore does not constitute independent confirmation of the factor structure.

## Table S4. Cohort- and semester-level baseline self-perceived AI literacy and pre-post effect sizes

n=138 (one response with an unparseable timestamp excluded). dz = mean change / SD of change.

### Panel A — by semester

| Semester | Months | Cohorts | n | Baseline mean (SD) | Post mean (SD) | dz |
| --- | --- | --- | --- | --- | --- | --- |
| 2024 Spring/Summer | Apr–May 2024 | 3 | 35 | 3.49 (0.56) | 4.25 (0.51) | 1.72 |
| 2024 Fall | Sep–Dec 2024 | 5 | 55 | 3.47 (0.78) | 4.08 (0.58) | 0.65 |
| 2025 Spring | Jan–May 2025 | 5 | 48 | 3.74 (0.49) | 4.40 (0.46) | 1.47 |

### Panel B — by session (cohort)

| # | Date | n | Baseline (SD) | Post (SD) | dz |
| --- | --- | --- | --- | --- | --- |
| 1 | 2024-04-01 | 12 | 3.37 (0.52) | 4.12 (0.40) | 1.49 |
| 2 | 2024-04-29 | 10 | 3.48 (0.67) | 4.15 (0.61) | 1.87 |
| 3 | 2024-05-13 | 13 | 3.62 (0.53) | 4.45 (0.50) | 1.82 |
| 4 | 2024-09-12 | 9 | 3.32 (0.72) | 4.07 (0.48) | 1.07 |
| 5 | 2024-09-26 | 12 | 3.73 (0.61) | 4.02 (0.61) | 0.65 |
| 6 | 2024-10-21 | 12 | 3.53 (0.86) | 4.07 (0.68) | 0.41 |
| 7 | 2024-11-04 | 10 | 3.49 (0.80) | 4.18 (0.71) | 0.76 |
| 8 | 2024-12-02 | 12 | 3.24 (0.90) | 4.05 (0.50) | 0.77 |
| 9 | 2025-01-17 | 9 | 3.62 (0.38) | 4.41 (0.45) | 1.96 |
| 10 | 2025-02-21 | 11 | 3.77 (0.46) | 4.32 (0.51) | 1.10 |
| 11 | 2025-03-21 | 6 | 3.91 (0.57) | 4.53 (0.57) | 2.32 |
| 12 | 2025-05-02 | 10 | 3.52 (0.70) | 4.32 (0.55) | 1.25 |
| 13 | 2025-05-16 | 12 | 3.91 (0.30) | 4.47 (0.32) | 1.89 |

Note. Pre-post gains were unrelated to cohort order (Spearman ρ=−0.07, P=.39), and baseline self-perceived AI literacy was not significantly associated with cohort order (ρ=0.17, P=.051; Kruskal–Wallis across cohorts P=.48). The PPOS-D6/CLAS subsample corresponds to the final five cohorts (2025).

## Table S5. PPOS-D6 score distribution and floor/ceiling (n=46)

Five-point scale (1–5); lower scores indicate greater patient-centered orientation. Floor = response of 1; ceiling = response of 5.

### Panel A — item level

| Item | Time | Mean (SD) | Skew | % at floor (1) | % at ceiling (5) |
| --- | --- | --- | --- | --- | --- |
| PPOS1 | Pre | 2.04 (0.97) | 0.69 | 32.6 | 0.0 |
| PPOS2 | Pre | 1.80 (0.58) | 0.03 | 28.3 | 0.0 |
| PPOS3 | Pre | 1.98 (0.93) | 0.91 | 34.8 | 2.2 |
| PPOS4 | Pre | 1.46 (0.69) | 1.65 | 63.0 | 0.0 |
| PPOS5 | Pre | 2.00 (0.97) | 1.08 | 32.6 | 2.2 |
| PPOS6 | Pre | 1.78 (0.96) | 1.08 | 50.0 | 0.0 |
| PPOS1 | Post | 1.87 (1.05) | 1.49 | 43.5 | 4.3 |
| PPOS2 | Post | 1.72 (0.83) | 1.54 | 45.7 | 2.2 |
| PPOS3 | Post | 2.04 (1.10) | 0.76 | 41.3 | 2.2 |
| PPOS4 | Post | 1.72 (0.98) | 1.64 | 52.2 | 2.2 |
| PPOS5 | Post | 2.02 (1.02) | 1.39 | 30.4 | 4.3 |
| PPOS6 | Post | 1.78 (1.03) | 1.48 | 50.0 | 2.2 |

### Panel B — total score (mean of 6 items)

| Time | Mean (SD) | Skew | Min | Max | % ≤1.5 (near floor) | % ≥4.5 (near ceiling) |
| --- | --- | --- | --- | --- | --- | --- |
| Pre | 1.84 (0.50) | −0.01 | 1.00 | 2.83 | 32.6 | 0.0 |
| Post | 1.86 (0.78) | 1.64 | 1.00 | 5.00 | 34.8 | 2.2 |

Note. At baseline, no participant scored above the scale midpoint (3.0) and responses occupied only ~46% of the available range, indicating a pronounced floor/restricted-range effect consistent with an already strongly patient-centered orientation at entry. This limits the instrument’s sensitivity to detect change and renders the non-significant pre-post comparison inconclusive on measurement grounds.
