# Supplementary material for: Integrating Generative AI Into Clinical Reasoning Education for Medical Students: Mixed Methods Study
Source: JMIR Med Educ. 2026 Aug 13;12:e94951. doi: 10.2196/94951 (PMC13472228; doi:10.2196/94951)
Supplement: Supplementary file 3 [file mededu-v12-e94951-s003.docx]

# **Completed GRAMMS (Good Reporting of a Mixed Methods Study) Checklist**

Reference: O’Cathain A, Murphy E, Nicholl J. The quality of mixed methods studies in health services research. *J Health Serv Res Policy*. 2008;13(2):92-98. doi:10.1258/jhsrp.2007.007074

| # | GRAMMS Criterion | Reported | Location in Manuscript | Note |
| --- | --- | --- | --- | --- |
| 1 | Describe the justification for using a mixed methods approach to the research question | Yes | Methods: Study Design (paragraph 1); Introduction (paragraph 5) | An explanatory sequential (sequential explanatory) mixed-methods design was chosen to capture both measurable learning outcomes (quantitative) and participants’ perspectives on the integration of generative AI into clinical reasoning education (qualitative). A quantitative pre-post outcome phase was followed by a qualitative phase that explains and contextualizes the quantitative findings. The rationale is stated in the Study Design subsection. |
| 2 | Describe the design in terms of the purpose, priority, and sequence of methods | Yes | Methods: Study Design; Data Collection; Data Analysis | Purpose: explanatory sequential (qualitative findings explain and contextualize the quantitative results). Priority: quantitative-dominant (QUAN → qual). Sequence: sequential, with quantitative scales administered pre-post, followed by qualitative interviews and reflections collected post-intervention; integration occurred at the interpretation and meta-inference level. |
| 3 | Describe each method in terms of sampling, data collection, and analysis | Yes | Methods: Setting and Participants; Data Collection; Data Analysis | Quantitative: 139 of 150 students provided paired pre-post responses on the AI literacy scale; the PPOS-D6 and CLAS were completed by a subsample of 46 students (final five cohorts, January–May 2025). All measures were analyzed with paired t-tests and Cohen’s d. Qualitative: 6 purposively sampled interview participants and 17 reflective writing participants; analyzed using reflexive thematic analysis following Braun and Clarke’s framework with independent dual coding. |
| 4 | Describe where integration has occurred, how it has occurred, and who has participated in it | Yes | Methods: Mixed Methods Integration; Results: Mixed Methods Integration; Discussion: Principal Results | Integration occurred at the interpretation and meta-inference level (described in dedicated Mixed Methods Integration subsections in both Methods and Results). Quantitative changes in self-perceived AI literacy and collaborative attitudes were examined alongside qualitative themes to identify three integrated learning dimensions: (1) technical AI literacy acquisition, (2) verification-oriented reasoning development, and (3) professional and ethical adaptation. All authors participated in the integration process. |
| 5 | Describe any limitation of one method associated with the presence of the other method | Yes | Discussion: Limitations | Limitations include: a single-group pre-post design without a comparator group, which limits causal inference for the quantitative findings; self-reported outcome measures, which may be subject to response-shift and social-desirability bias; an adapted and translated instrument whose four conceptual domains were highly intercorrelated and showed only modest confirmatory-factor-analysis fit, so the four-factor structure is treated descriptively rather than as formal cross-cultural validation; a reduced and not fully representative subsample (n = 46) for PPOS-D6 and CLAS that limited statistical power (the study could detect only medium-or-larger PPOS-D6 change, minimum detectable dz = 0.42) and showed a baseline floor effect, rendering the null PPOS-D6 result inconclusive; possible secular changes in AI exposure over the 13-month enrollment; and qualitative sample sizes guided by information power rather than a fixed saturation threshold. |
| 6 | Describe any insights gained from mixing or integrating methods | Yes | Results: Mixed Methods Integration; Discussion (all subsections); Conclusions | Integration of methods revealed three interconnected dimensions of learning not captured by either method alone. Qualitative data identified mechanisms underlying quantitative changes (e.g., evolving GenAI understanding supported self-perceived AI literacy gains; the non-significant PPOS-D6 result was interpreted as inconclusive given a baseline floor effect and limited statistical power, rather than as stable patient-centered orientation; prompt-engineering skill progression contextualized Application domain improvements). The emergent theme of professional identity reconceptualization was discovered exclusively through qualitative data and enriched the overall interpretation. Integration enabled the formulation of three curriculum design dimensions and four design principles for GenAI-integrated medical education. |
